# Structural and optical properties of germanium nanostructures on Si(100) and embedded in high-k oxides

**DOI:** 10.1186/1556-276X-6-224

**Published:** 2011-03-15

**Authors:** Samit K Ray, Samaresh Das, Raj K Singha, Santanu Manna, Achintya Dhar

**Affiliations:** 1 Department of Physics and Meteorology, Indian Institute of Technology Kharagpur 721302, India

## Abstract

The structural and optical properties of Ge quantum dots (QDs) grown on Si(001) for mid-infrared photodetector and Ge nanocrystals embedded in oxide matrices for floating gate memory devices are presented. The infrared photoluminescence (PL) signal from Ge islands has been studied at a low temperature. The temperature- and bias-dependent photocurrent spectra of a capped Si/SiGe/Si(001) QDs infrared photodetector device are presented. The properties of Ge nanocrystals of different size and density embedded in high-k matrices grown using radio frequency magnetron sputtering have been studied. Transmission electron micrographs have revealed the formation of isolated spherical Ge nanocrystals in high-k oxide matrix of sizes ranging from 4 to 18 nm. Embedded nanocrystals in high band gap oxides have been found to act as discrete trapping sites for exchanging charge carriers with the conduction channel by direct tunneling that is desired for applications in floating gate memory devices.

## Introduction

Germanium nanostructures have potential applications for electronic flash memories [1-3] and light emitters in visible [4] and near-infrared [5] wavelengths, making the indirect gap semiconductor attractive for novel electronic and optical devices. In comparison to bulk Ge, nanocrystals exhibit a tunable emission wavelength [6] and increased oscillator strength due to the quantum confinement of excitons. The confinement of charge carriers in these nanostructures allows one to increase the efficiency of the radiative recombination. The growth of Ge islands on Si substrates via Stranski-Krastanow growth mode has been extensively investigated as this opens up the possibility to integrate optoelectronics with planar Si technology. Most of the SiGe/Si structures are believed to exhibit a type-II heterointerface, where electrons and holes are spatially separated with a limited wave function overlap [7]. Owing to the type-II band alignment, Ge quantum dots (QDs) themselves form a potential well only for holes, whereas the electrons are weakly confined in their vicinities, i.e., by the tensile and compressive strain fields in the Si cap induced by the strained QDs [8]. This has led to the enhancement of PL quantum efficiency in planar Si/SiGe superlattices at elevated temperatures due to 3D carrier localization within the Ge QDs and presumably due to large energy barriers formed at the heterointerfaces between the Ge clusters and the surrounding Si matrix [9].

On the other hand, intersubband transitions in Ge/Si quantum dots (QDs) are attractive for quantum dot infrared photodetectors (QDIPs) in the wavelength range 5-10 μm. Ge QDIPs have an advantage that the absorption of normally incident infrared radiation by holes in the valence band is allowed, without the requirement of fabrication of gratings or any other optical coupling elements unlike for the conduction band of III-V semiconductors. Similarly, persistent efforts have been made to achieve efficient visible light emission from Si and Ge nanocrystals (NCs) embedded in oxide matrix [10]. Even though Ge NCs embedded in the high band gap oxide matrix show efficient and tunable PL emission by varying their size, the origin of the light emission is still under debate [3,6].

In this article, we report the structural and optical properties of Ge QDs grown on Si(001) by molecular beam epitaxy (MBE) as well as Ge nanocrystals embedded in high band gap oxide matrices. The observed infrared PL signal from Ge dots grown on Si(001) is influenced by island size and the intermixing of Si/Ge. The origin of photoresponse of the Ge islands in the mid-infrared (IR) wavelength range is discussed. The emission and charge trapping behavior of Ge nanocrystals embedded in different high band gap oxide matrices are also reported.

## Experimental

Ge QDs on Si(001) substrates were grown by solid source MBE (Riber Supra 32) system using an electron gun for the deposition of thin Si buffer layer (approximately 5 nm) with a growth rate of 0.4 Å/s and a Knudsen cell for Ge deposition, followed by the growth of a 3.0 nm Si cap layer. Growth temperature was varied from 500 to 600°C and Ge monolayer (ML) thickness was assorted from 6 to 20 ML. The growth was monitored in situ by reflection high energy electron diffraction (RHEED). On the other hand, Ge nanocrystals embedded in high-k HfO_2 _and Al_2_O_3 _matrix on Si(100) substrates were deposited by radio frequency (13.56 MHz) magnetron co-sputtering method in Ar + O_2 _ambient at an rf power of 50 W, similar to those reported earlier [2,3]. The as-grown sample is defined as 'A-as' and 'F-as' for Ge embedded Al_2_O_3 _and HfO_2_, respectively. In order to grow Ge nanocrystals in high-k matrix, the sputter deposited film was thermally annealed in N_2 _gas ambient for 30 min at 800 and 900°C.

The growth of Ge islands using MBE was studied using Veeco, Nanoscope-IV atomic force microscope (AFM). High-resolution transmission electron microscopy (HRTEM) was carried out using a JEM 2100F (JEOL) field emission system with an operating voltage of 200 kV to probe the formation of Ge nanocrystals in the oxide matrix. Raman spectra of the grown samples were obtained with a Renishaw Raman microscope equipped with a He-Ne laser excitation source emitting at a wavelength of 632.8 nm and a Peltier cooled (-70°C) charge-coupled device (CCD) camera. PL spectra of samples were recorded using a He-Cd laser as an excitation source, operating at 325 nm with an output power density of 1.3 W/cm^2 ^and a TRIAX 320 monochromator fitted with a photomultiplier or an InGaAs detector. The photocurrent (PC) spectra were investigated under monochromatic light dispersed from a glowbar source by grating spectrometer and chopped at a frequency of 233 Hz. PC signals were detected by a standard lock-in amplifier technique. Aluminum was deposited on top of the sample by masking and was rapid thermal annealed at 200 C for 10 min to form a good ohmic contact. At backside, native oxide was etched by HF followed by Al deposition to form the ohmic contact. The electrical properties of the grown structures were measured using a Keithley semiconductor parameter analyzer (4200-SCS).

## Results and discussion

Growth and optical properties of Ge nano-islands on Si(001)

**Figure 1:**
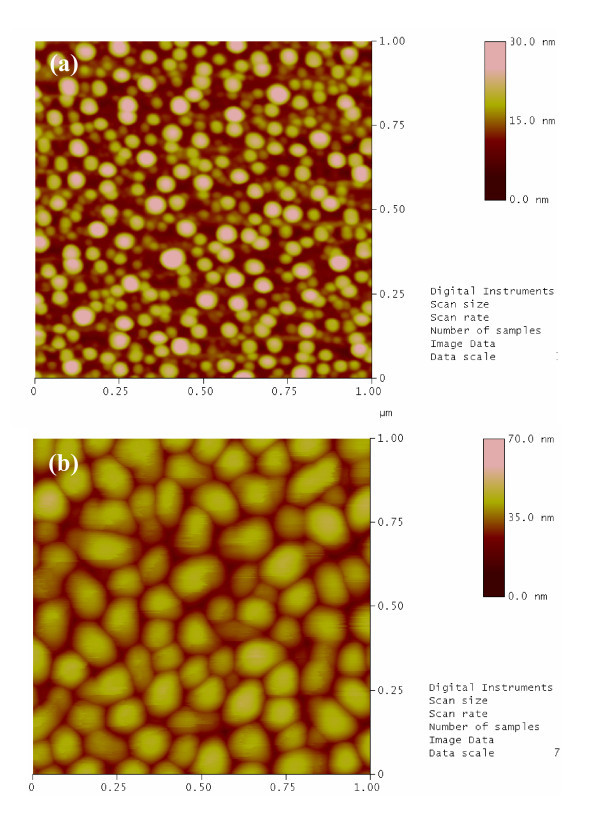
**Typical AFM topographic images for (a) 2 min (sample 'GS-1') and (b) 5 min (sample 'GS-2') grown Ge islands deposited at a substrate temperature 500°C**.

Figure 1a, b shows the AFM images of the MBE grown Ge islands deposited for 2 (sample 'GS-1') and 5 min (sample 'GS-2'), respectively, at a substrate temperature of 500°C. From AFM topographic images, the variation of island shape, size, and density is clearly visible. A bimodal size distribution of islands is visible from Figure 1a. The average diameter (*L*), height (*h*) for larger and smaller islands are *L *= 54 nm, *h *~ 18 nm and *L *= 23 nm, *h *= 7 nm, respectively, for sample 'GS-1'. On the other hand, the growth of multifaceted dome like structure is evident in Figure 1b for the 'GS-2' sample with the average islands size *L *= 90 nm and *h *= 35 nm. In Stranski-Krastanov (S-K) growth of above islands, the arrangement of deposited Ge atoms begins with the formation of a strained planar layer called the wetting layer (WL), until a critical thickness is reached. A further increase of the deposited material leads to the nucleation of three-dimensional Ge islands on the wetting layer. At the first stage of the growth, the islands are square-based pyramids [11,12]. Upon collecting more adatoms by the process of coarsening (Ostwald ripening) from neighboring islands, these pyramids transform to strained multifaceted domes. From Figure 1b it is seen that for longer time Ge deposition, the smaller islands coalesce to form multifaceted domes. One can also change the islands size and shape distribution by post-growth annealing [13].

**Figure 2:**
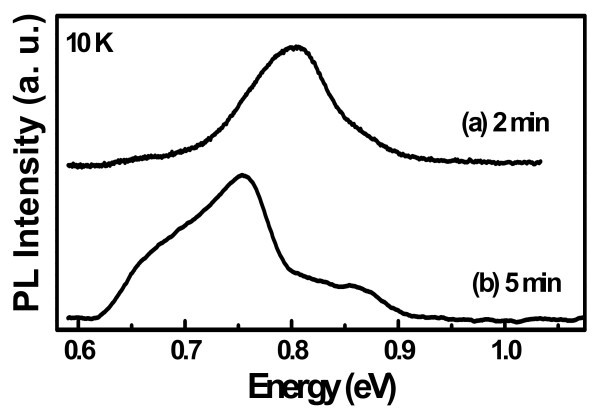
**10 K photoluminescence spectra of Ge islands grown on Si substrate for sample (a) GS-1 and (b) GS-2**.

The strength of no-phonon transitions and the overlap of electron and hole wave functions can be enhanced in Si/Ge nanostructures, but their quantum efficiency remains orders of magnitude below that of direct optical transitions. Figure 2 shows the 10 K PL spectra of self-assembled Ge QDs grown at 500°C for (a) 2 min (sample 'GS-1') and (b) 5 min (sample 'GS-2'). Broad PL peaks are observed around 0.755 and 0.804 eV for samples grown for 5 and 2 min, respectively. The observed broad PL signal from Ge/Si islands is associated with the radiative carrier recombination at sharp Ge/Si interface that exhibits type-II band alignment, with a small barrier for electrons and deep potential wells for the holes confined within the Ge islands [9]. Due to lower height (7 to 18 nm) of the islands, the PL peak of 2 min sample is blue shifted compared to sample grown for 5 min. Another cause for the shift may be due to the intermixing of Si and Ge for longer time (5 min) deposition of Ge, which reduces the band offset between islands and Si interface.

**Figure 3:**
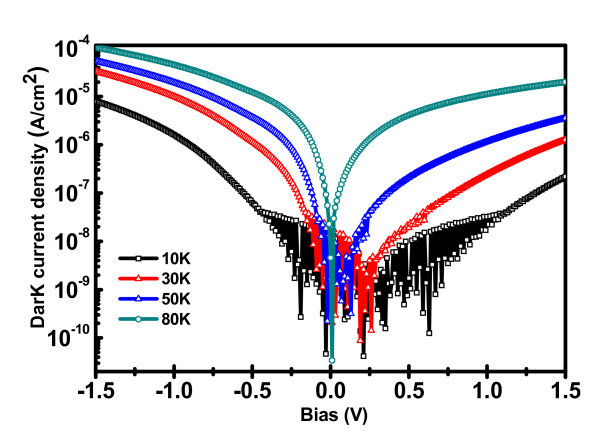
**The dark current density-voltage characteristics of a Ge QDIP structure measured at low temperatures**.

The photo-response of Ge QD infrared photodetector (QDIP) has been studied at varying temperatures. Figure 3 shows the temperature-dependent dark current-voltage (*J*-*V*) characteristics of the QDIP device. The dark current density at 10 K is lower as compared to conventional infrared photo-detectors. The dark current density increases at elevated temperatures due to thermionic emissions. The fluctuations in *J-V *characteristic in both bias directions at 10 K are clearly observed, which reduces with increasing temperature and dies out at 40 K. This phenomenon is attributed to the carrier localization at the Si/Si_1-*x*_Ge*_x _*hetero-interface. These localized carriers result in an in-built voltage (*V*_b_) varying from 0.2 to 0.32 V from 10 to 300 K. One origin of this carrier localization may be due to the confined holes and large valance band offset in Ge/Si heterostructure in type-II band alignment [14], which results in well for electrons at the Ge/Si interface in Si.

**Figure 4:**
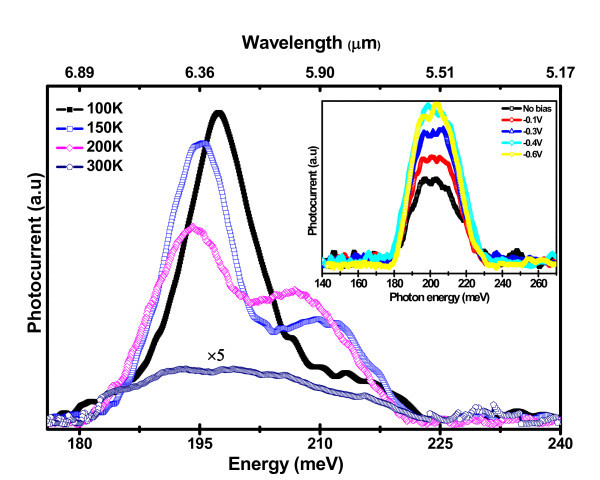
**The mid-IR photocurrent spectra of capped Ge/Si QDs at different temperatures**. The inset curve shows the photocurrent at room temperature under different reverse bias conditions.

Low temperature PC response was measured using a closed cycle cryostat with KBr window. The mid-IR (180-220 meV) PC response of the grown Ge QDs in the temperature range 100-300 K is shown in Figure 4 at zero applied bias. The mid-IR peak at 195 meV is redshifted with increasing temperature up to 175 K. Although the maximum PC response is observed at 175 Ka shoulder peak at (205 meV) is evolved with increasing temperature, which exhibits a redshift up to 175 K. At room temperature these two peaks merge to yield a broader response. The curves in the inset of Figure 4 show the mid-IR PC response at room temperature under different -ve bias voltages. The peak intensity increases with applied -ve bias and saturates at -0.6 V. The PC saturates when no further holes can be pumped out from the confined energy states with increasing bias. The redshift arising in PC on increasing the temperature up to 175 K is not due to Stark effect, since no peak shift is observed by applying external electrical field in both bias polarities at low and room temperatures. The observed redshift and peak PC response can be explained by the excitonic electric field localized at the interface developed at low Ge nanocrystals embedded in high-k matrices.

**Figure 5:**
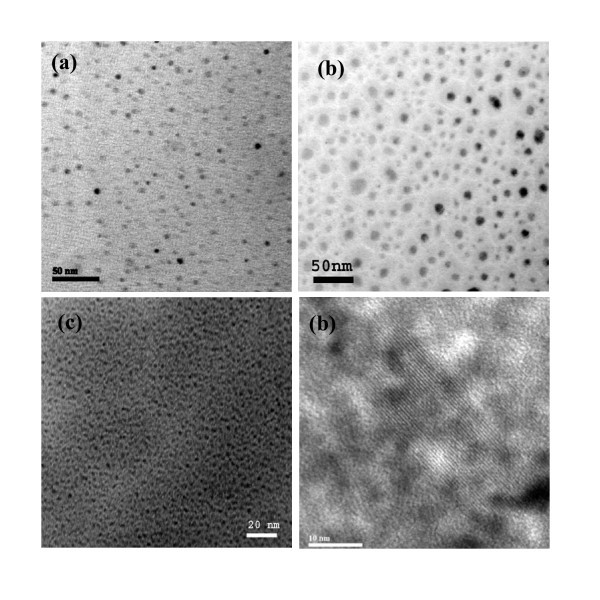
**Plan-view TEM micrograph of Ge NCs embedded in high-k matrix for (a) A-800, (b) A-900, (c) F-800, and (d) F-900 samples**.

Figure 5a, b shows the plane-view TEM images of Ge NCs embedded in Al_2_O_3 _and annealed at 800 and 900°C, respectively. The samples are hereafter referred as A-800 and A-900, respectively. The dark patches seen are Ge nanocrystals embedded in amorphous Al_2_O_3 _matrix. The nanocrystals are almost spherical and are well dispersed in the host matrix. The estimated size distribution of the nanocrystals for A-800 sample can be approximated by a Gaussian distribution with an average diameter of 7.6 nm. For A-900 sample, the distribution of the nanocrystals throughout the film is not uniform and the diameter varies from 9 to 17 nm. Figure 5c, d shows the plane-view TEM images of Ge NCs embedded in HfO_2 _and annealed at 800°C (sample F-800) and 900°C (sample F-900), respectively. The image resolution in Figure 5d for F-900 sample is comparatively higher. The average diameter of the Ge NCs for F-800 sample is about 3.9 nm, whereas for F-900 sample it varies from 7 to 13 nm. The change in Gibbs free energy of formation of GeO (111.8 kcal/mol) is much smaller than that of high-k oxides, such as HfO_2 _(260.1 kcal/mol) and Al_2_O_3 _(378.2 kcal/mol) [15], which results in the oxidation of Hf or Al and agglomeration of Ge atoms into nanocrystals in HfO_2 _or Al_2_O_3 _matrix during thermal annealing at high temperatures. It is observed that when annealed at 800°C, which is well below the melting temperature of Ge (938.3°C), only Ge nucleation occurs. Whereas for both 900°C annealed samples (A-900 and F-900), Ge nanocrystals usually show nonuniform distribution of size and density within high-k oxide matrix due to the high diffusion rate of Ge atoms, in consistent with the previously reported results [16]. Furthermore, a higher annealing temperature is expected to result in increased critical nucleus size.

**Figure 6:**
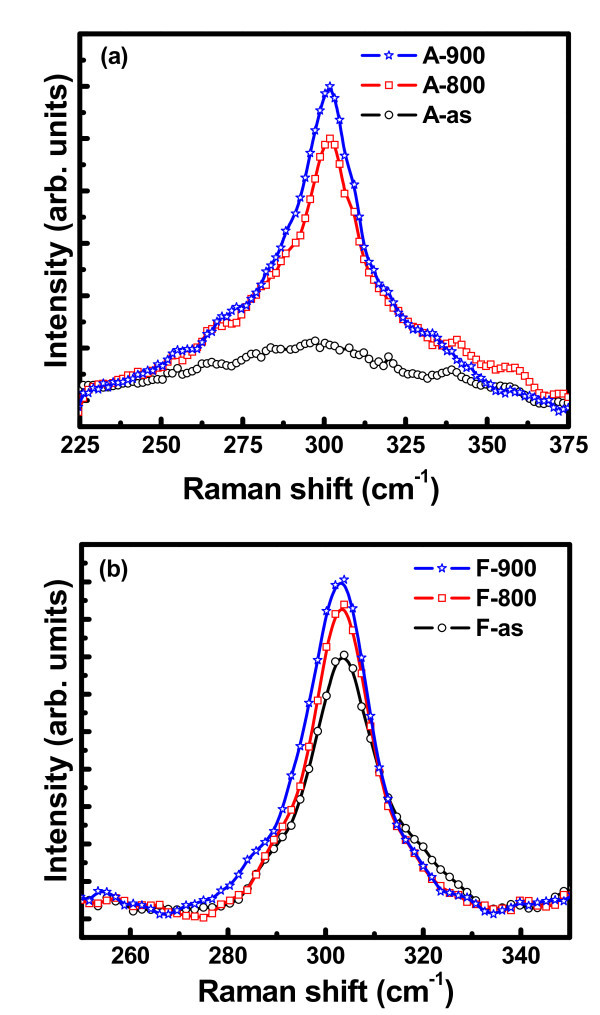
**Raman spectra of Ge nanocrystals embedded in (a) Al_2_O_3 _and (b) HfO_2 _matrix in the as-grown and annealed state**.

Quantitative structural examinations of Ge nanocrystals have been carried out using Raman spectroscopy. Figure 6a, b shows the Raman spectra of Ge nanocrystals embedded in Al_2_O_3 _and HfO_2 _matrix, respectively, in the as-grown state and after post-growth thermal annealing at 800 and 900 C for 30 min in N_2 _atmosphere. Raman spectra of nanocrystals are characterized by the size-dependent phonon confinement effects which, for the case of Si and Ge, are manifested by asymmetric line broadening and a red shift of the peak due to breakdown of the *k *= 0 selection rule for Stokes scattering. The Raman peak at around 300 cm^-1 ^is attributed to the crystalline Ge-Ge phonon vibration mode, indicating the formation of Ge nanocrystals. A blue shift of Raman spectra of silica-embedded [17], sapphire-embedded [18], and Hafnia-embedded [19] Ge NCs has been reported, which has been attributed to the matrix-induced compressive stress on embedded nanocrystals. This blue shift of the peak position with respect to that of the bulk reference spectrum is in disagreement with the prediction of phonon confinement theory. The stress may also arise due to the volumetric expansion of Ge during solidification [17], fast growth rate experienced by nanocrystals as a result of enhanced diffusivity [20] and from the interface energy. The hydrostatic pressure P in the nanocrsytals can be estimated as [21],(3)

where *ω*_stressed_, *ω*_relaxed_, and *ω*_0 _are the Raman shifts of stressed NCs embedded in HfO_2_, relaxed NCs, and pure bulk Ge, respectively; *γ *= 0.89 [20] is the mode-Grüneisen parameter, and S_11 _and S_12 _are components of the elastic compliance tensor with S_11 _+ 2S_12 _= 0.44 × 10^-12 ^dyne^-1 ^cm^2 ^[21]. Since suitable selective etchant for Al_2_O_3 _and HfO_2 _is not available; the relaxed line position has been calculated [19] using the phonon confinement model developed by Richter et al. [22]. The calculated Raman peaks for relaxed Ge using phonon confinement model [22,19] in comparison to the experimental values for embedded Ge nanocrystals in different high-k matrices are summarized in Table 1. The hydrostatic pressure *P *calculated using the above values from Equation 3 for different samples is also presented in Table 1. From Figure 6a, b it is observed that the intensity of Ge-Ge phonon peak increases with the post-deposition annealing temperature due to an increase in the Ge concentration in the film on reduction of germanium suboxides concentration. Thus the formation of Ge nanocrystals most likely originates from the dissociation of suboxides and agglomeration of Ge during N_2 _annealing.

**Table 1:** Raman peak for relaxed (phonon confinement model) and embedded (experimental) Ge nanocrystals and estimated hydrostatics stress

Sample	Nanocrystal distribution			Ge-Ge phonon peak position		Stress
	Diameter (nm)	FWHM (nm)	Density (cm-2)	Experimental (embedded) (cm-1)	Phonon confinement (relaxed) (cm-1)	P (GPa)

A-800	7.1	2.6	3.5 × 1011	301.5	297.4	1.2
A-900	13	6	2.6 × 1011	301.2	298.4	0.8
F-800	3.9	2.1	5.8 × 1012	303.3	294.2	2.6
F-900	10	4	1.3 × 1012	302.7	297.9	1.4

**Figure 7:**
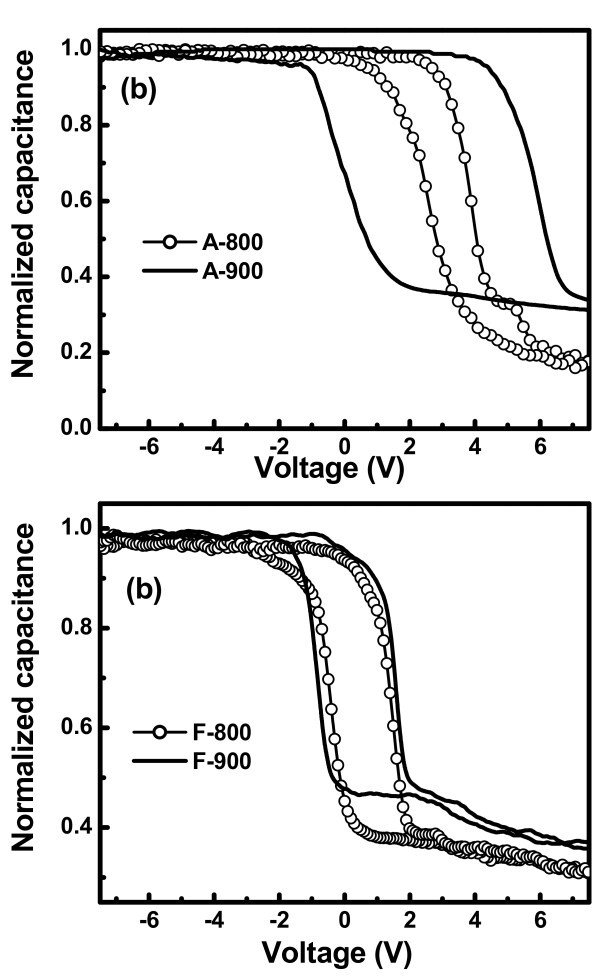
**High-frequency (100 kHz) C-V characteristics of MOS capacitor with Ge nanocrystals embedded in high-k (a) Al_2_O_3 _and (b) HfO_2 _matrix**.

Figure 7a, b represents the high-frequency (100 kHz) capacitance-voltage (C-V) hysteresis behavior of the MOS structures fabricated using Ge nanocrystals embedded in Al_2_O_3 _and HfO_2 _matrix, respectively, for a voltage sweep of ± 7.5 V. A negligibly small flat-band voltage shift of 0.15 and 0.18 V is observed for Al_2_O_3 _and HfO_2 _MOS devices without Ge NCs. However, a large memory window of 1.20, 6.32, 1.85, and 2.38 V is observed for the A-800, A-900, F-800, and F-900 samples, respectively. From the maximum flat-band voltage shift of the C-V curves, we have calculated the stored charge density using the following equation [23](4)

where Δ*V*_FB _is the flat-band voltage shift, *q *is the magnitude of the electronic charge; *t*_CO _and *ε*_CO _are the thickness and relative permittivity of the control oxide; *t*_NC _and *ε*_NC _are the diameter and relative permittivity of the nanocrystal; and *ε*_O _is the permittivity of the free space. The calculated stored charge densities for the A-800, A-900, F-800, and F-900 devices are 1.3 × 10^12^, 7.1 × 10^12^, 4.5 × 10^12^, and 5.4 × 10^12 ^cm^-2^, respectively. Comparing with the nanocrystal density of the above samples presented in Table 1, it is evident that the numbers of charges stored per nanocrystal are around 4, 27, 1, and 4 for the samples A-800, A-900, F-800, and F-900, respectively. For sample F-900, with average nanocrystal diameter 3.9 nm, the charge stored per nanocrystal is one due to prominent Coulomb blockade effect in small clusters. Whereas for other samples with larger diameter, there are more than one electron per nanocrystal due to reduced Coulomb repulsion. The number of stored charges per cluster is highest for the sample A-900 with largest size (13 nm). The memory window and stored charge density is found to be significantly enhanced on increasing the annealing temperature (900°C) for Ge nanocrystals embedded in Al_2_O_3 _matrix as compared to that of HfO_2_, making it attractive for nanocrystal flash memory applications.

**Figure 8:**
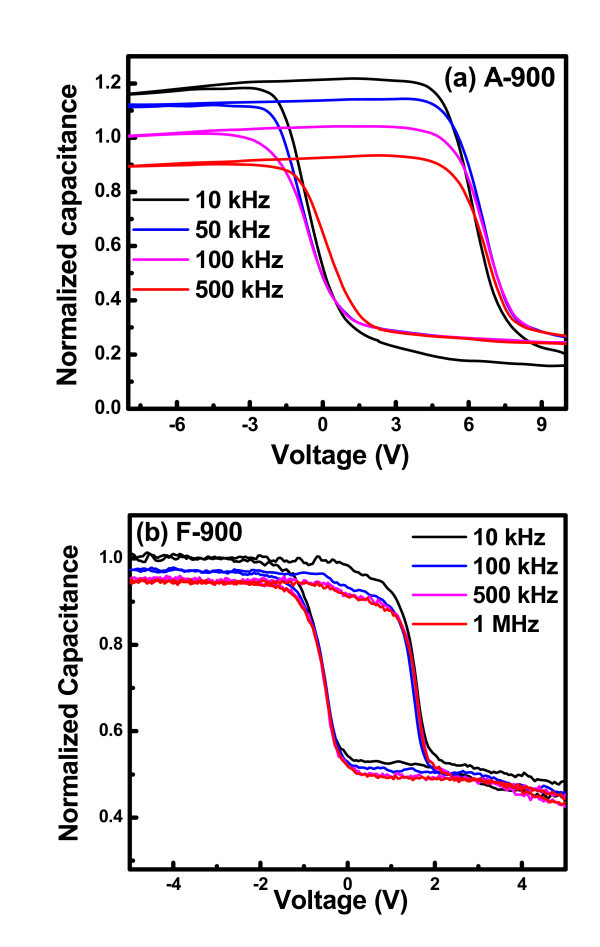
**Frequency-dependent C-V characteristics at room temperature for the samples (a) A-900 and (b) F-900**.

The origin of C-V hysteresis can be accredited either to injected charges mainly in nanocrystals or at the interfaces between the NCs and the surrounding oxides. To understand the contribution of trapped charges in detail, the frequency-dependent capacitance-voltage measurement has been carried out for the samples annealed at 900°C. Figure 8a, b shows the frequency dependent C-V curves for the samples A-900 and F-900 for ± 10 and ± 6 V sweep voltages, respectively. Almost similar anti-clockwise C-V hysteresis in the frequency range from 10 kHz to 1 MHz was observed with no stretch-out along the gate voltage axis for the entire experimental frequency range at room temperature. This indicates that the hysteresis is not due to interface traps, as they generally give rise to frequency-dependent flat-band shift and stretching of C-V characteristics [24].

## Conclusions

We have presented the structural and optical characteristics of Ge islands grown on Si(100) by MBE. The observed infrared PL signal at 10 K from Ge islands is associated with the radiative recombination of holes confined in the Ge islands and electrons localized in the Si buffer layer. The temperature and bias dependent PC spectra of a capped Si/SiGe/Si(001) QDIP photodetector device are presented. We have also grown Ge nanocrystals (4-18 nm in diameter) embedded in high-k Al_2_O_3 _and HfO_2 _matrices for applications in floating gate memory devices. The analysis of Ge-Ge phonon vibration using Raman spectroscopy has shown the formation of compressively stressed Ge nanocrystals in high-k matrix. The observed shift in flat-band voltage for C-V curves has been attributed to electron trapping in embedded Ge nanocrystals.

## Abbreviations

AFM: atomic force microscope; CCD: charge-coupled device; HRTEM: high-resolution transmission electron microscopy; IR: infrared; MBE: molecular beam epitaxy; ML: monolayer; NCs: nanocrystals; PC: photocurrent; PL: photoluminescence; QDs: quantum dots; QDIP: quantum dot infrared photodetector; RHEED: reflection high energy electron diffraction; WL: wetting layer.

## Competing interests

The authors declare that they have no competing interests.

## Authors' contributions

SD, RKS and SM carried out all the experiments. SD and RKS performed the analysis of experimental data and calculations. SD and SKR prepared the manuscript initially. SKR and AD conceived of the study and participated in its design and coordination. All authors read and approved the final manuscript.
